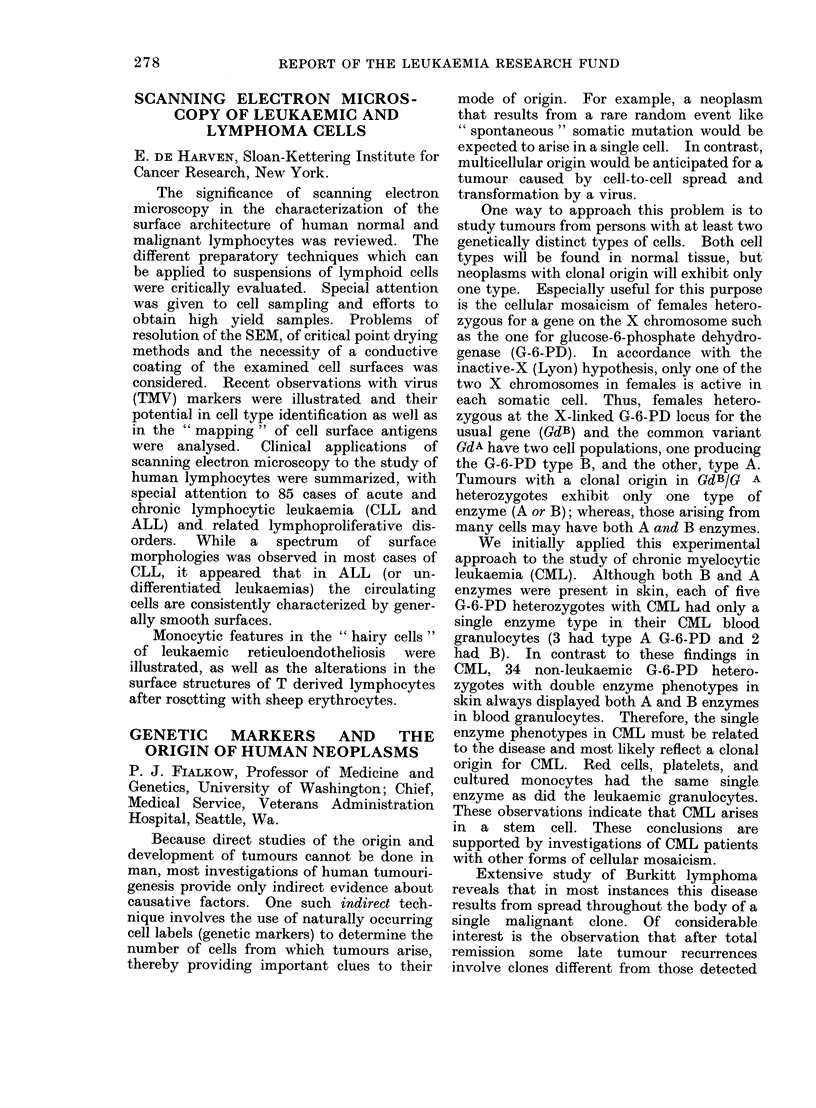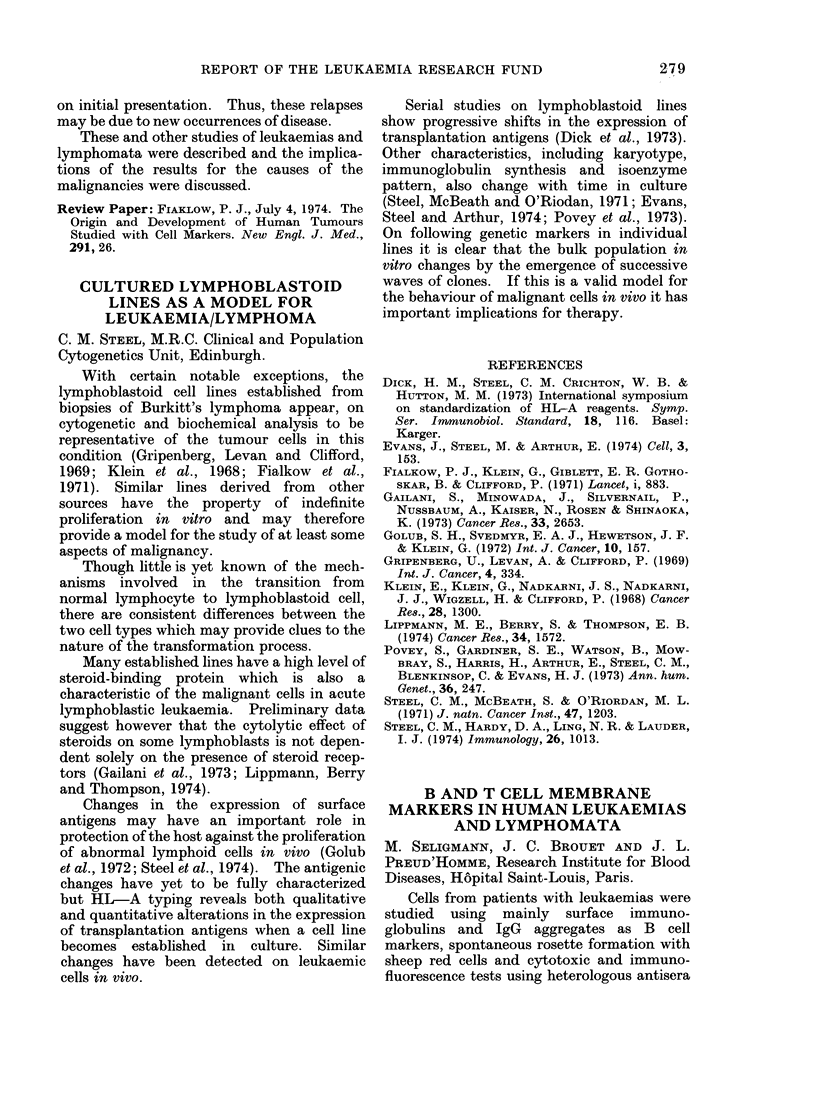# Proceedings: Genetic markers and the origin of human neoplasms.

**DOI:** 10.1038/bjc.1975.215

**Published:** 1975-08

**Authors:** P. J. Fialkow


### GENETIC MARKERS AND THE ORIGIN OF HUMAN NEOPLASMS

P. J. FIALKOW, Professor of Medicine and Genetics, University of Washington; Chief, Medical Service, Veterans Administration Hospital, Seattle, Wa.

Because direct studies of the origin and development of tumours cannot be done in man, most investigations of human tumourigenesis provide only indirect evidence about causative factors. One such *indirect* technique involves the use of naturally occurring cell labels (genetic markers) to determine the number of cells from which tumours arise, thereby providing important clues to their mode of origin. For example, a neoplasm that results from a rare random event like " spontaneous " somatic mutation would be expected to arise in a single cell. In contrast, multicellular origin would be anticipated for a tumour caused by cell-to-cell spread and transformation by a virus.

One way to approach this problem is to study tumours from persons with at least two genetically distinct types of cells. Both cell types will be found in normal tissue, but neoplasms with clonal origin will exhibit only one type. Especially useful for this purpose is the cellular mosaicism of females heterozygous for a gene on the X chromosome such as the one for glucose-6-phosphate dehydrogenase (G-6-PD). In accordance with the inactive-X (Lyon) hypothesis, only one of the two X chromosomes in females is active in each somatic cell. Thus, females heterozygous at the X-linked G-6-PD locus for the usual gene ($Gd^{B}$) and the common variant $Gd^{A}$ have two cell populations, one producing the G-6-PD type B, and the other, type A. Tumours with a clonal origin in $Gd^{B}/G^{A}$ heterozygotes exhibit only one type of enzyme (A *or* B); whereas, those arising from many cells may have both A *and* B enzymes.

We initially applied this experimental approach to the study of chronic myelocytic leukaemia (CML). Although both B and A enzymes were present in skin, each of five G-6-PD heterozygotes with CML had only a single enzyme type in their CML blood granulocytes (3 had type A G-6-PD and 2 had B). In contrast to these findings in CML, 34 non-leukaemic G-6-PD heterozygotes with double enzyme phenotypes in skin always displayed both A and B enzymes in blood granulocytes. Therefore, the single enzyme phenotypes in CML must be related to the disease and most likely reflect a clonal origin for CML. Red cells, platelets, and cultured monocytes had the same single enzyme as did the leukaemic granulocytes. These observations indicate that CML arises in a stem cell. These conclusions are supported by investigations of CML patients with other forms of cellular mosaicism.

Extensive study of Burkitt lymphoma reveals that in most instances this disease results from spread throughout the body of a single malignant clone. Of considerable interest is the observation that after total remission some late tumour recurrences involve clones different from those detected on initial presentation. Thus, these relapses may be due to new occurrences of disease.

These and other studies of leukaemias and lymphomata were described and the implications of the results for the causes of the malignancies were discussed.

**Review Paper:** FIAKLOW, P. J., July 4, 1974. The Origin and Development of Human Tumours Studied with Cell Markers. *New Engl. J. Med.*, **291**, 26.